# “Platelet-Rich Plasma” epidural injection an emerging strategy in lumbar disc herniation: a Randomized Controlled Trial

**DOI:** 10.1186/s12891-023-06429-3

**Published:** 2023-04-28

**Authors:** Asarn Wongjarupong, Saran Pairuchvej, Panyajarn Laohapornsvan, Vit Kotheeranurak, Khanathip Jitpakdee, Chunrutai Yeekian, Pongsthorn Chanplakorn

**Affiliations:** 1 Department of Orthopaedic, Queen Savang Vadhana Memorial Hospital, Siracha, Chonburi, Thailand; 2 Department of orthopedic, Faculty of medicine Ramathibodi Hospital, Mahidol University, Bangkok, Thailand

**Keywords:** ESI, Lumbar HNP, Platelet-rich plasma, PRP, Triamcinolone, VAS

## Abstract

**Background:**

Lumbar herniated disc (HNP) is mainly treated by conservative management. Epidural steroid injection (ESI) has been an option to treat failed cases prior to surgery. Triamcinolone has been widely used due to its efficacy in bringing about pain reduction for up to three months. However, several reports have shown some severe adverse events. Platelet-rich plasma (PRP) is made from blood through centrifugation. Several studies supported the potential short to long-term effects, and safety of PRP injection in treating HNP. The study objective was to evaluate the efficacy of PRP in treatment of single-level lumbar HNP in comparison to triamcinolone.

**Methods:**

Thirty patients were treated by transforaminal epidural injections. PRP was obtained from 24 ml venous blood through standardized double-spin protocol. Participants included fifteen patients each being in triamcinolone and PRP groups. The same postoperative protocols and medications were applied. The visual analogue scale of leg (LegVAS), collected at baseline, 2, 6, 12, and 24 weeks, was the primary outcome. The BackVAS, Oswestry Disability Index (ODI), adverse event, and treatment failure were the secondary endpoints.

**Results:**

Platelet ratio of PRP in fifteen patients was 2.86 ± 0.85. Patients treated by PRP injections showed statistically and clinically significant reduction in LegVAS at 6, 12, and 24 weeks, and in ODI at 24 weeks. It demonstrated comparable results on other aspects. No adverse event occurred in either group.

**Conclusion:**

Noncommercial epidural double-spin PRP yielded superior results to triamcinolone. Due to its efficacy and safety, the procedure is recommended in treating single level lumbar HNP.

**Trial registration:**

NCT, NCT05234840. Registered 1 January 2019, https://clinicaltrials.gov/ct2/show/record/NCT05234840.

**Supplementary Information:**

The online version contains supplementary material available at 10.1186/s12891-023-06429-3.

## Introduction

Lumbar radicular pain is usually caused by mechanical compression of nerve root, and by inflammatory responses [1–3]. Conservative management, such as rest, physical therapy, and oral medications, is the mainstay in treating lumbar herniated disc. However, only 70% had significant pain reduction, and up to 20% had recurrent symptoms [4]. Transforaminal epidural steroid injection (ESI) has been an option to reduce the radicular pain prior to the surgery [5, 6]. The therapeutic agent, triamcinolone, has been widely used due to its anti-inflammatory effect, and low complication. However, several reports have shown some severe adverse effects such as infection, allergic reaction, and endocrine suppression [7, 8]. Most randomized studies have demonstrated significant improvement in pain relief in the first three months. However, controversial outcomes have shown up in pain reduction of long-term follow-up and rate of necessitating later operation at one year [9–12].

Platelet-rich plasma (PRP) has recently gained a name as an adjuvant component in the orthopedics field [13]. PRP property depends on platelet concentration, white blood cell concentration and activation [14]. PRP contains numerous cytokines and growth factors, including Interleukin-1 receptor antagonist (IL-1Ra), Transforming Growth Factor- β1 (TGFβ-1), Platelet-Derived Growth Factor (PDGF) and Insulin-like Growth Factor-1 (IGF-1) [15]. According to its autologous and antimicrobial property, PRP provides minimal risks in immunogenic reactions, side effects, and surgical site infection [16]. Main mechanisms were anti-inflammatory and neural regeneration pathway, and disc resorption. Several studies supported potential the short, to long-term effects and safety of PRP and platelet-rich product in treating HNP [17–20].

The study aims were to compare the results of transforaminal PRP injection and traditional ESI on lumbar radicular leg pain by VAS (LegVAS), back pain (BackVAS), functional score (Oswestry Disability Index, ODI), adverse event, and percentage of treatment failure in HNP patient.

## Materials and methods

### Study design

The study was a triple blinded, randomized controlled trial in level I referral center. The study was conducted between April 2019 to May 2021. After the assessment of eligibility was accomplished. The inclusion criteria were patients aged 20–55 years, having failed conservative treatment of unilateral HNP undergone for at least 6 weeks, with visual analogue scale (VAS) of greater than 30, and confirmed a single-level HNP, corelated to clinical, by MRI. The exclusion criteria included previous spine surgery or epidural injection, progressive neurological deficit, cauda equina, coagulopathy-related conditions, associated cervical myelopathy, systemic bone and joint diseases. All patients had full conservative management including rest, activity modification, oral medication, and physical therapy by rehabilitation team. Patients with no improvement in VAS and greater than 30 were defined as failed conservative management. All patients had to have been exempted from NSAIDs for at least one week. The study was approved by the Ethics Committee of Queen Savang Vadhana Memorial Hospital (QSVMH, 015/2563), and receipt of the ClinicalTrials.gov ID was NCT05234840. All participants provided informed consent.

Based on the non-inferiority formula, **n= [(r + 1)(Z**_**1−**β_+**Z**_**1−**α_**)**^**2**^σ^**2**^**]/r[(**µ_**A**_**-**µ_**B)−**_**d**_**NI**_**]**^**2**^ was used. Previous similar study’s data were used for this calculation [20]. Together with a power of 80% (β = 0.20), and a level of significant of 5% (α = 0.05), fifteen patients per group were needed. Eligible patients were randomized by the four-block method in 1:1 allocation ratio. Details of patients enrolled into the study were shown in Table 1. Randomization was done by a computer-generated program on the appointment date. Results were sealed in envelopes which were opened just after blood was obtained from the patient by assisting staff. Blood tubes in the PRP group were then sent to the preparation room.

### PRP preparation [21]

1. 26 ml of blood was obtained from each patient (6ml each in 4 CPDA (citrate phosphate dextrose adenine) and 2ml in EDTA (ethylene diamine tetra acetic acid tube) for CBC).
2. First-spin of 900 g for 5 min (Kokusan, H-19alpha, 25 °C).
3. Three layers formed: transfer top layer (platelet poor plasma) and middle layer (platelets and WBC) to another sterile tube (about 3.3 ml), discard bottom layer (RBC).
4. Second-spin of 1000 g for 10 min.
5. The upper one third was discarded (platelet poor plasma) by pipet, the remaining was mixed by turning 10 times.
6. Final product was 4 ml of PRP (3ml for procedure, 1ml for cell differentiation and culture).

**Fig. 1:**
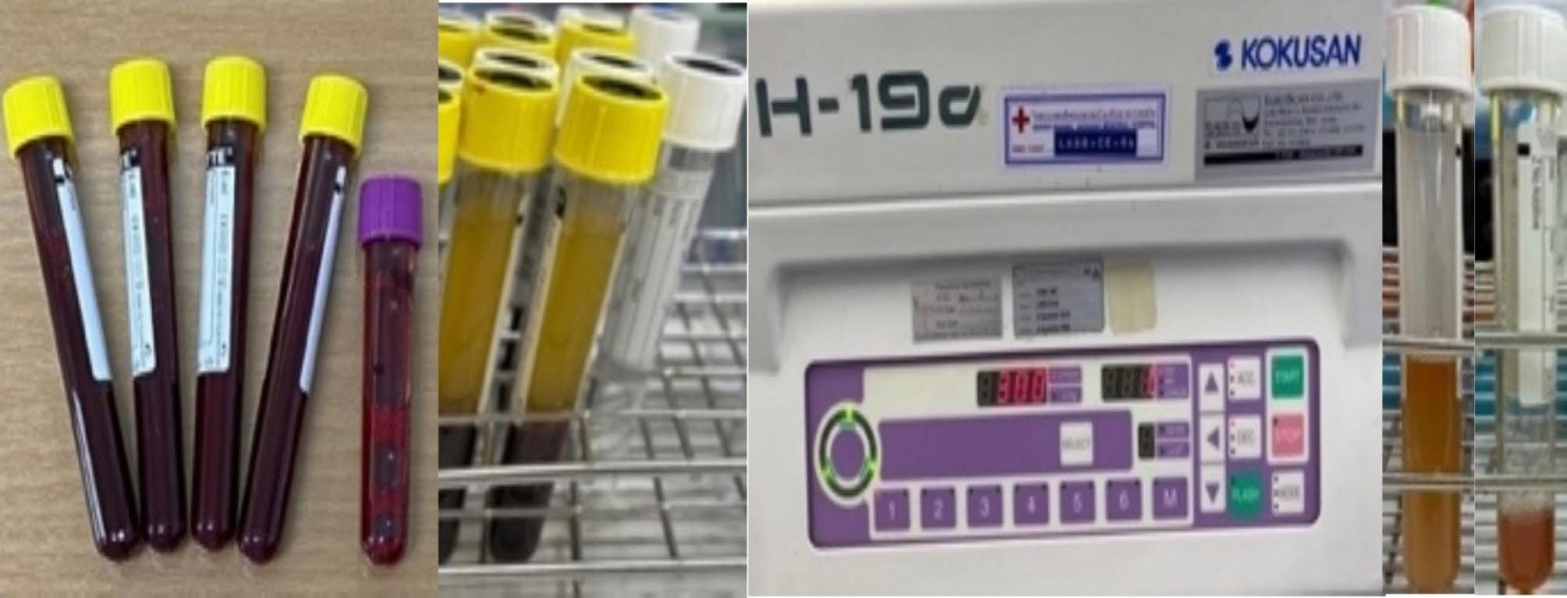
Four tubes of CPDA were collected with one EDTA tube. First-spin product. Machine used for PRP preparation. Second-spin product. 3 ml of PRP for transforaminal epidural injection

### Transforaminal epidural injection

All treatments were performed by a single, experienced orthopedic surgeon. Patients were placed in the prone position. Using a sterile technique, epidural injections were performed under a C-arm fluoroscopy (Phillps, USA). Briefly, the transforaminal approach through Kambin’s triangle was used to minimize risk of nerve injury. The position of the needle (Quincke Chiba Needle, 22-gauge: 8 inch), was guided and confirmed under anteroposterior and lateral fluoroscopic views. Once the needle-end was located and checked by contrast media, either 2 mL of PRP followed by NSS 0.5 ml, or total of 2ml of 1% lidocaine with 40 mg triamcinolone was injected. The syringe was covered with a large sterile strip to mask the substance. The patient was observed for 30 min after the procedure to monitor for any undesirable reaction. All patients were asked to avoid strenuous activities in the ensuing 3 days. Cold compression and paracetamol were provided for pain relief.

**Fig. 2:**
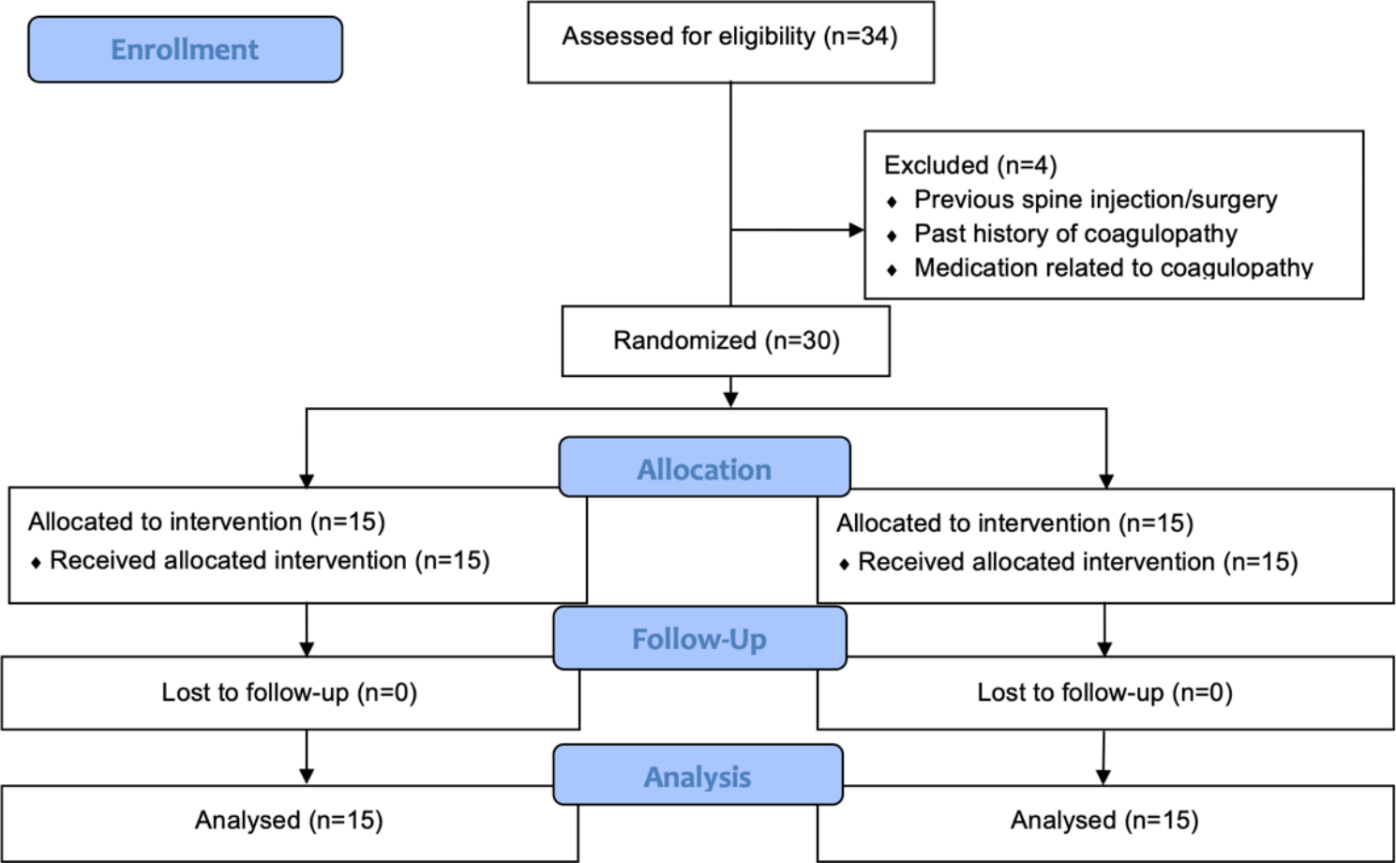
Consort diagram

### Evaluation and follow-up

Primary outcome was radicular leg pain evaluated by LegVAS. A diagram was presented to the patient with a score of 0 denoting “no pain”, and a score of 100 denoting “pain as bad as it could be.” BackVAS, ODI adverse event, and failure of treatment were also addressed at baseline, 2, 6, 12, 24 weeks. Patients were observed for acute postoperative complications for at least 4 to 6 h. Medication prescribed was paracetamol and tolperisone. All patients used ice pack for 1 day and lumbar support for 3 days after the injection. Patients were advised of acute complications such as soreness, itching, neurological deficits, and infection. Limited back activities were recommended for 1 week. There was no drop out, and no cross-over group. Treatment failure was defined as persistent or deteriorated pain. MRI was requested when there was persistent pain or new onset of pain.

### Statistical analysis

An independent t-test, Fisher’s Exact test, and Chi-square test were used for demographic data, presented as mean ± SD, and percentage. Repeated ANOVA was used to compare the mean of quantitative variables over time. The Chi-square test was used to assess failure of treatment. The p value < 0.05 was considered statistically significant. Data were analyzed with STATA 17. All study parameters were analyzed according to the intention-to-treat (ITT) principle.

## Results

A total of 30 patients (15 patients in each group) were enrolled in this study. No significant differences were found in the demographic data, as shown in Table 1. From the protocol described previously [21], the overall platelet concentration were exceeding 10 lakhs/ml. In our study, the platelet ratio was 2.85 ± 0.85 (mean ± SD) with hematocrit 2.25 ± 1.20. The PRP contained WBC 12,610 ± 4202 cells/ml. The Lymphocyte was the dominant type, 75.35 ± 5.92%.

**Table 1:** Demographic Variables

Variables	Triamcinolone (n = 15) Mean ± SD	**PRP (n = 15)** Mean ± SD	*p-value*
	Mean ± SD	Mean ± SD	
**Age (year)**	39.13 ± 7.21	39.73 ± 7.04	0.819
**Weight**	68.33 ± 13.95	72.93 ± 12.09	0.343
**Height**	163.2 ± 9.41	161.93 ± 7.64	0.689
**BMI (kg/m** ^**2**^ **)**	25.55 ± 4.15	27.89 ± 4.88	0.167
**Gender female**	7 (46.7%)	6 (40%)	0.723
**Disc type**			0.208
**Bulging**	9 (60%)	5 (33.3%)	
**Protrusion**	2 (13.3%)	6 (40%)	
**Extrusion**	4 (26.7%)	4 (26.7%)	
**Disc location**			0.855
**Central**	6 (40%)	5 (33.3)	
**Paracentral**	9 (60%)	9 (60%)	
**Foraminal**	0 (0%)	1 (0.7%)	
**Level**			0.715
**L4/5**	7 (46.7%)	8 (53.3%)	
**L5/S1**	8 (53.3%)	7 (46.7%)	
**Onset (month)**	3.47 ± 1.6	3 ± 1	0.34
**LegVAS pre**	73 ± 13.86	64.27 ± 17.53	0.141
**BackVAS pre**	59.67 ± 19.68	65.27 ± 15.32	0.392
**ODI pre**	43.13 ± 10.81	44.73 ± 10.71	0.686

Independent t test, Fisher’s Exact test, and Chi-square test

**Fig. 3:**
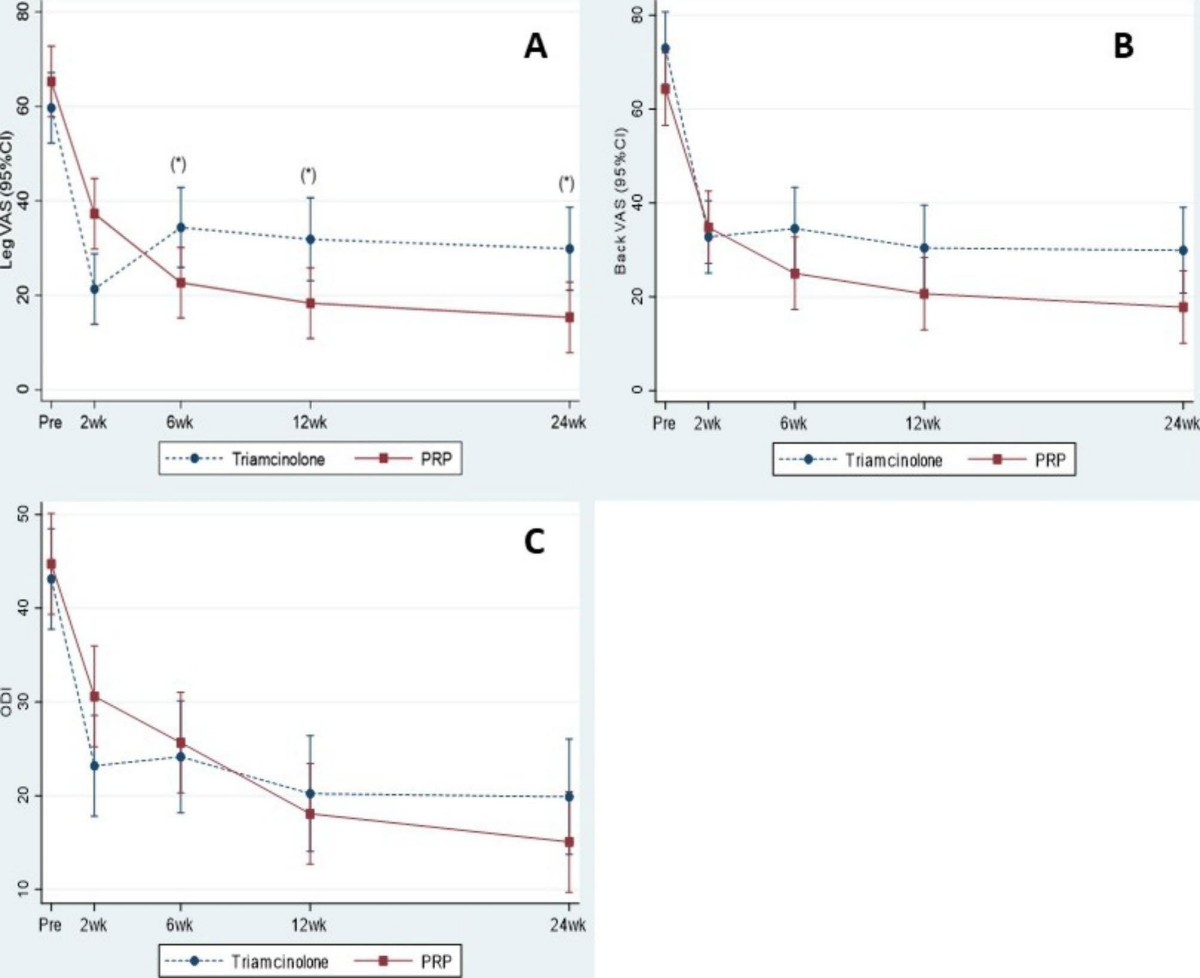
The change in LegVAS (**A**), BackVAS (**B**) and ODI (**C**) between the 2 treatment groups; triamcinolone and PRP. The results are shown as mean ± SD. Data were analyzed with repeated ANOVA with p-value < 0.05

According previous studies [22], the value of 15 and 5 were used as minimal clinical important difference (MCID) of VAS and ODI, respectively. LegVAS reduction was both statistically and clinically significant at 6 to 24 weeks. BackVAS reduction was not statistically significant but showed comparable results. ODI only showed clinical significance at 24 weeks, without statistical significance (Table 2). Figures 1, 2 and 3 show the trend of changes in LegVAS, BackVAS, and ODI at various time-point.

**Table 2:** LegVAS, BackVAS, and ODI

LegVAS	Triamcinolone (n = 15)	Mean change (95%CI)	PRP (n = 15)	Mean change (95%CI)	Mean difference Between groups (95%CI)	p-value
Baseline	59.67 ± 19.68	Reference	65.27 ± 15.32	Reference	Reference	1
2wk	21.33 ± 16.85	-38.33 (-47.02, -29.65)	37.27 ± 19.03	-28 (-35.91, -20.09)	10.33 (-1.35, 22.02)	0.083
6wk	36.36 ± 16.9	-25.19 (-34.82, -15.56)	22.67 ± 15.34	-42.6 (-50.51, -34.69)	-17.29 (-29.64, -4.95)	0.006*
12wk	32 ± 11.35	-27.78 (-37.72, -17.83)	18.33 ± 13.58	-46.93 (-54.84, -39.02)	-19.13 (-31.7, -6.56)	0.003*
24wk	30 ± 7.45	-29.78 (-39.72, -19.83)	15.33 ± 9.9	-49.93 (-57.84, -42.02)	-20.13 (-32.7, -7.56)	0.002*
**Back VAS**	**Triamcinolone (n = 15)**	**Mean change** **(95%CI)**	**PRP** **(n = 15)**	**Mean change** **(95%CI)**	**Mean difference** **Between groups (95%CI)**	**p-value**
Baseline	73 ± 13.86	Reference	64.27 ± 17.53	Reference	Reference	1
2wk	32.8 ± 24.84	-40.2 (-50.26, -30.14)	34.8 ± 19.37	-29.47 (-36.84, -22.1)	10.73 (-1.52, 22.99)	0.086
6wk	35 ± 17.18	-38.36 (-49.48, -27.25)	25 ± 13.09	-39.27 (-46.64, -31.9)	-0.82 (-13.76, 12.12)	0.901
12wk	29 ± 13.08	-42.78 (-54.26, -31.31)	20.67 ± 8.84	-43.6 (-50.97, -36.23)	-1.02 (-14.19, 12.16)	0.88
24wk	28.5 ± 10.29	-43.28 (-54.76, -31.81)	17.8 ± 11.33	-46.47 (-53.84, -39.1)	-3.38 (-16.56, 9.8)	0.615
**ODI**	**Triamcinolone (n = 15)**	**Mean change** **(95%CI)**	**PRP** **(n = 15)**	**Mean change** **(95%CI)**	**Mean difference** **Between groups (95%CI)**	**p-value**
Baseline	43.13 ± 10.81	Reference	44.73 ± 10.71	Reference	Reference	1
2wk	23.19 ± 15.84	-19.93 (-25.95, -13.92)	30.6 ± 12.56	-14.13 (-18.77, -9.5)	5.8 (-1.69, 13.29)	0.129
6wk	27 ± 13.3	-18.73 (-25.42, -12.03)	25.67 ± 11.61	-19.07 (-23.7, -14.43)	-0.09 (-8.03, 7.84)	0.981
12wk	21.64 ± 7.92	-22.75 (-29.67, -15.83)	18.07 ± 8.06	-26.67 (-31.3, -22.03)	-3.77 (-11.85, 4.32)	0.361
24wk	21.3 ± 5.21	-23.09 (-30.01, -16.17)	15.07 ± 7.79	-29.67 (-34.3, -25.03)	-6.42 (-14.51, 1.66)	0.120

**Table 3:** Treatment failure

Variables	Triamcinolone (n = 15)	PRP (n = 15)	*p-value*
**Yes**	2 (13.3%)	0 (0%)	0.143
**No**	13 (86.7%)	15 (100%)	

Chi-square test

No adverse events such as local inflammation, infection, and neurological deficit occurred in either group. Two patients in triamcinolone group needed later operation due to persistent pain at 6 weeks follow-up. There was no statistical difference in treatment failure between the two groups (Table 3).

## Discussion

Prolotherapy uses a method of inflammatory induction to recruit cytokines and growth factors, which later promote healing cascade. However, the rationale of using PRP is it contains high concentration of cytokines and growth factors which are released overtime [15]. The other advantages of PRP are autologous product and antimicrobial properties.

PRP usage has been studied in orthopedic spine surgery, mainly in treating degenerative disc disease. Prior to PRP usage, Becker’s RCT reported superior results of PRP to triamcinolone on LegVAS by using of Autologous Conditioned Serum (ACS), another type of orthobiologics, in treating HNP by multiple injections [20]. Recently, interlaminar and transforaminal epidural injections were used to treat radicular pain from herniated disc with various substance; ACS [23], platelet-rich in growth factor (PRGF) [24], PRP [17, 18], and plasma lysate [19]. The outcomes of leg pain reduction were effective up to three months. One study reported significant pain reduction and disability improvement up to six months in twenty patients [23]. The safety of epidural injection with plasma lysate was conducted with 470 registries, also demonstrating promising results in VAS, functional rating index (FRI), and a modified single assessment numeric evaluation (SANE) up to two years follow up [19]. Cemeron also reported effectiveness of PRP in HNP treatment with the improvement of VAS up to 77%, and ODI 8.7% in 8 years follow-up [18]. Our study showed superior results of PRP to triamcinolone on LegVAS at 6,12 and 24 week. This might be from reparative and regenerative effect of PRP [28, 29]. In our study protocol of PRP preparation, we achieved high platelet concentration which contained a large number of growth factors and cytokines in such case yielded a better effect of anti-inflammatory, reparative and regenerative effect.

Patients treated with PRP showed significant improvement in VAS, ODI, and other functional scores. PRP showed high potential in regeneration and healing via several mechanism such as inflammatory regulation pathway, cellular stimulation, and tissue regeneration pathway in both animal model and human [25]. PRP are rich in growth factors including IL-1Ra, Transforming Growth Factor- β1 (TGFβ-1), Platelet-Derived Growth Factor (PDGF) and Insulin-like Growth Factor-1 (IGF-1). The two main mechanisms were postulated were anti-inflammatory and neural regeneration pathway, and disc resorption. The anti-inflammatory pathway is to reduce the neural inflammation via IL-1Ra by attach to IL-1 to inhibit inflammatory stimulation [20, 26, 31] and TGFβ-1 signaling to block initiation and maintenance of inflammation [27]. The neural regeneration was through combination effects of PDGF and IGF-1 in regeneration of peripheral nerve and myelin [28, 29] as mentioned by Centeno [19]. The disc resorption is via macrophage-induced phagocytosis stimulated by growth factors.

According to the meta-analysis about spontaneous disc resorption, the overall incidence is about 67% in conservative patients [30]. Moreover, previous case report showed significant decreased size of herniated disc as found in our study [26]. However, Our study had only 2 cases of post-injection MRI which was insufficient to conclude the effect of decreasing disc size significantly.

We concurred with Zhen Xu et al.’s study on efficacy of PRP compared to betamethasone in treating lumbar HNP in term of pain score and functional score [32]. Our study demonstrated better outcome for PRP for leg VAS over cortisone at 6,12 and 24 week. These difference might be from variation of platelet concentration and amount of betamethasone (which was not mentioned) in the study. Further studies are required to summarize the efficacy of PRP over steroid in term of chronic leg pain.

Our research conducted RCT, with similar demographic data with single experience surgeon, represented an emerging substitute, PRP, in treating single-level HNP. The drawback for PRP is injection time which is within one hour after final product. PRP production protocol takes about 30 min for overall process. This protocol can be done in any hospital because it requires simple centrifuge with easy-to-follow protocol and laboratory staff with sterile technique due to an open-system. Our limitations were short time follow-up, subjective primary outcome as VAS, and too small sample size to detect the statistically significant of treatment failure.

## Conclusion

The present study showed that alternative PRP injection provided a superior result to triamcinolone on LegVAS, and comparable results on BackVAS, ODI, adverse event, and treatment failure. The prolonged and superior effect with potentially lower rate of treatment failure supports the usage of PRP in HNP treatment. Noncommercial PRP protocol is safe, reproducible and effective in the treatment of lumbar HNP. We encourage using PRP protocol [21], instead of commercial kit.

## Electronic supplementary material

Below is the link to the electronic supplementary material.

Supplementary Material 1

## Data Availability

The datasets used and analyzed during the current study are available from the corresponding author on reasonable request.

## List of Abbreviations

ESI: Transforaminal epidural steroid injection
PRP: Platelet-rich plasma
IL-1Ra: Interleukin-1 receptor antagonist
TGFβ-1: Transforming Growth Factor- β1
PDGF: Platelet-Derived Growth Factor
IGF-1: Insulin-like Growth Factor-1
HNP: Lumbar herniated disc
VAS: Visual analog scale
ODI: Oswestry Disability Index
CPDA: Citrate phosphate dextrose adenine
EDTA: Ethylene diamine tetra acetic acid
NSS: Normal saline
MCID: Minimal clinical important difference
ACS: Autologous Conditioned Serum
PRGF: Platelet-rich in growth factor
FRI: Functional rating index
SANE: Single assessment numeric evaluation
IGF-1: Insulin-like Growth Factor-1
