# Supplementary figures and images for: “Platelet-Rich Plasma” epidural injection an emerging strategy in lumbar disc herniation: a Randomized Controlled Trial
Source: BMC Musculoskelet Disord. 2023 Apr 28;24:335. doi: 10.1186/s12891-023-06429-3 (PMC10141936; doi:10.1186/s12891-023-06429-3)

**Supplementary material**

**PRP data**

**
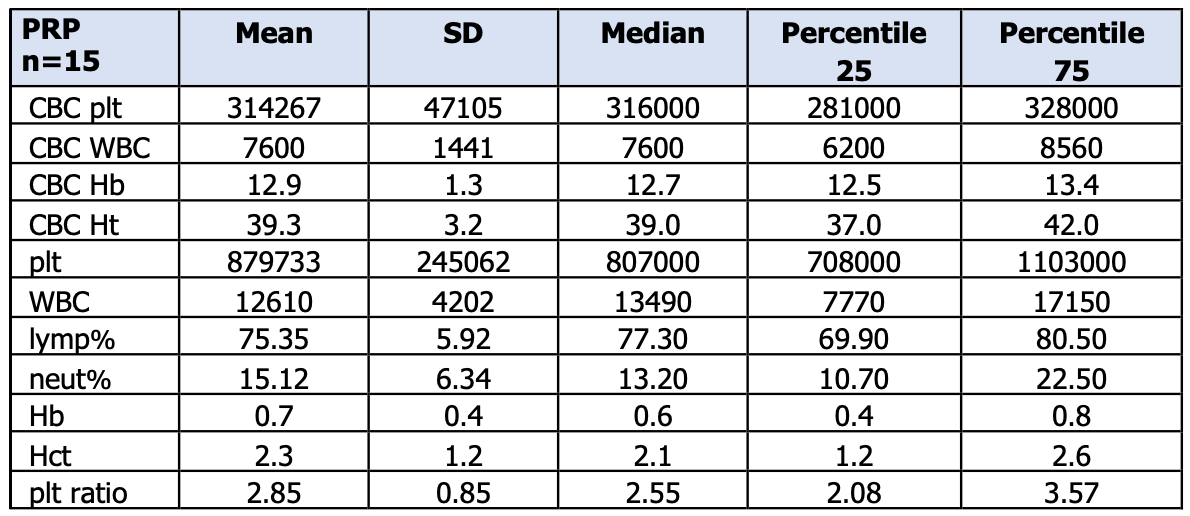
**

Supplement: Supplementary file 1 — Supplementary Material 1 [file 12891_2023_6429_MOESM1_ESM.docx]
